# Triglyceride-glucose index and nocturnal oxygenation impairment in patients with obstructive sleep apnea

**DOI:** 10.3389/fendo.2026.1899452

**Published:** 2026-07-02

**Authors:** Chong Pei, Lingli Hao, Jingjing Zhang, Kang Xu, Yiqiong Yu, Lei Hu

**Affiliations:** 1 Department of Pulmonary and Critical Care Medicine, The Third Affiliated Hospital of Anhui Medical University (Hefei First People’s Hospital), Hefei, Anhui, China; 2 Department of Gastroenterology, The Third Affiliated Hospital of Anhui Medical University (Hefei First People’s Hospital), Hefei, Anhui, China; 3 Department of Ultrasound Medicine, The First Affiliated Hospital of USTC, Division of Life Sciences and Medicine, University of Science and Technology of China (Anhui Provincial Hospital), Hefei, Anhui, China

**Keywords:** CT90, insulin resistance, lowest oxygen saturation, nocturnal oxygenation impairment, obstructive sleep apnea, triglyceride-glucose index

## Abstract

**Background:**

In obstructive sleep apnea (OSA), respiratory events differ not only in frequency but also in the depth and duration of oxygen desaturation. The triglyceride-glucose (TyG) index is a routinely available marker related to insulin resistance, but its relationship with conventional nocturnal oxygenation-related parameters in OSA remains incompletely clarified. Objective: We examined the association of the TyG index with hypoxemia-related sleep parameters and explored whether these associations were influenced by monitoring modality and AHI-defined OSA severity.

**Methods:**

This single-center retrospective cross-sectional study screened 154 consecutive symptomatic adult inpatients from 2021 to 2025; after exclusion of 11 patients, 143 were included in the retrospective dataset. Diagnostic sleep monitoring was performed using in-laboratory polysomnography or Nox T3 portable monitoring. Laboratory measurements were obtained within 3 days before or after sleep monitoring. Severe nocturnal oxygen desaturation was defined as lowest SpO_2_ <80%, and severe AHI-defined OSA was defined as AHI ≥30 events/h.

**Results:**

TyG was available in 116 patients, and 101 had complete TyG, lowest SpO_2_, and AHI data. TyG was higher in patients with lowest SpO_2_ <80% than in those with lowest SpO_2_ ≥80% [9.252 (8.872, 9.577) vs. 8.780 (8.574, 8.963), P<0.001], and higher in patients with AHI ≥30 events/h than in those with AHI <30 events/h [9.242 (8.869, 9.624) vs. 8.823 (8.571, 9.121), P<0.001]. TyG correlated positively with AHI and CT90 and negatively with mean and lowest SpO_2_. The AUC was 0.760 for lowest SpO_2_ <80% and 0.715 for AHI ≥30 events/h. In models adjusted for age, sex, BMI, and monitoring modality, TyG remained associated with lowest SpO_2_ <80% and AHI ≥30 events/h. However, the association with lowest SpO_2_ <80% was attenuated after additional adjustment for AHI.

**Conclusion:**

Higher TyG was associated with worse nocturnal oxygenation-related parameters and greater AHI-defined OSA severity, but these exploratory findings may partly reflect overall OSA severity. TyG may provide a simple metabolic clue for risk stratification, but it cannot replace formal sleep monitoring.

## 1 Introduction

Obstructive sleep apnea (OSA) is a common sleep-related breathing disorder characterized by recurrent partial or complete upper-airway obstruction during sleep, resulting in intermittent hypoxemia, sleep fragmentation, and sympathetic activation (1–3). Its clinical importance extends beyond snoring and daytime sleepiness, as OSA is closely linked to hypertension, abnormal glucose metabolism, dyslipidemia, and cardiovascular disease (4–6).

The apnea-hypopnea index (AHI) remains the standard metric for diagnosing OSA and grading disease severity. However, AHI primarily captures the frequency of respiratory events and provides limited information about the depth, duration, and cumulative impact of oxygen desaturation. Conventional oxygenation parameters, including mean oxygen saturation, lowest oxygen saturation, time spent below 90% oxygen saturation (CT90/T90), and oxygen desaturation indices, can therefore provide complementary information to AHI (7, 8). More advanced sleep-apnea-specific hypoxic burden metrics have also been increasingly emphasized because they may better reflect physiological stress and cardiometabolic risk than event frequency alone (9, 10).

Metabolic dysfunction is another important feature of OSA. Recurrent nocturnal oxygenation impairment may contribute to oxidative stress, inflammation, sympathetic activation, and adipose tissue dysfunction, all of which can worsen glucose-lipid metabolism (6, 11). The triglyceride-glucose (TyG) index, derived from fasting triglyceride and glucose concentrations, is an inexpensive surrogate marker related to insulin resistance and has been validated against direct measures of insulin sensitivity (12–14).

Several previous studies have linked the TyG index or TyG-related indices with OSA risk and AHI-defined severity. For example, population-based and clinical studies have reported that higher TyG is associated with increased OSA risk or greater AHI-defined severity (15–20), and a recent systematic review also supported an association between TyG and OSA (21). However, most available studies focused mainly on OSA diagnosis, risk, or AHI-based severity, while less attention has been paid to conventional nocturnal oxygenation parameters, such as lowest SpO_2_ and CT90. This distinction is clinically relevant because oxygenation-related indices may capture aspects of OSA-related physiological stress that are not fully reflected by AHI.

In this exploratory retrospective study, we examined the association between the TyG index and nocturnal oxygenation-related parameters in hospitalized patients with OSA-related evaluation. We also assessed whether TyG was associated with marked nocturnal oxygen desaturation and severe AHI-defined OSA, and performed additional sensitivity analyses to account for monitoring modality and overall OSA severity.

## 2 Materials and methods

### 2.1 Study design and participants

We conducted a single-center retrospective cross-sectional study. Consecutive symptomatic adult inpatients admitted to The Third Affiliated Hospital of Anhui Medical University (Hefei First People’s Hospital) from 2021 to 2025 were reviewed. Patients were identified from the hospital information system if they were hospitalized for typical OSA-related symptoms, including habitual snoring, witnessed apnea, nocturnal gasping or choking, non-restorative sleep, or excessive daytime sleepiness, and underwent OSA-related evaluation during hospitalization.

A total of 154 consecutive symptomatic adult inpatients were screened. After excluding 3 patients with a history of malignancy and 8 patients who had previously received continuous positive airway pressure treatment and were readmitted for follow-up care, 143 patients were included in the retrospective dataset (**Supplementary Figure 1**). Patients were eligible for the analytic cohort if they were at least 18 years old and had valid in-hospital diagnostic sleep-monitoring data sufficient to determine AHI and oxygenation parameters. Patients without valid in-hospital diagnostic sleep-monitoring data, including those with only pressure-titration records or outside-hospital diagnostic sleep-study results unavailable in the electronic record, were excluded from analyses requiring AHI or oxygenation outcomes.

Exclusion criteria were severely incomplete core data, severe acute infection, malignancy, severe hepatic or renal insufficiency, acute cardiovascular or cerebrovascular events, pregnancy, and prior continuous positive airway pressure or other OSA treatment that could have altered sleep-monitoring results. Analyses for each endpoint used complete cases with available TyG and the corresponding sleep outcome.

The Medical Ethics Committee of Hefei First People’s Hospital approved the study (approval number: 2026-134-01). Written informed consent was waived because the study was retrospective, used de-identified clinical data, and involved minimal risk to participants.

### 2.2 Sleep monitoring and scoring

In-hospital sleep monitoring was performed using either in-laboratory polysomnography (PSG; Philips Alice 6 system) or a portable sleep-monitoring system (Nox T3 Sleep Monitor). In the retrospective dataset, monitoring modality was coded as PSG, Nox T3 portable monitoring, or no valid in-hospital diagnostic monitoring. PSG and Nox T3 monitoring are both objective sleep-monitoring approaches used in clinical practice. PSG is the gold standard for adult OSA diagnosis, whereas home or portable sleep apnea testing can be used in clinically suspected moderate-to-severe uncomplicated OSA when clinically appropriate (1, 22–24).

Recorded channels included airflow, thoracoabdominal movement, SpO_2_, pulse, snoring, body position, EEG, EMG, and ECG signals. Because both PSG and Nox T3 recordings included EEG channels, sleep time was objectively estimated and AHI was calculated based on total sleep time in both modalities rather than recording time. Respiratory events were first generated by the device software and then manually reviewed and corrected by trained sleep technicians. Raw sleep-monitoring signals were manually inspected, and obvious respiratory or oximetry artifacts, including signal dropout or poor-quality oximetry signals, were excluded before analysis. The minimum acceptable valid recording duration was 4 hours. Hypopneas were scored using a 4% oxygen desaturation criterion, consistent with accepted sleep-scoring approaches (25).

Sleep-monitoring variables extracted from the electronic records included AHI, mean SpO_2_, lowest SpO_2_, CT90, and longest apnea duration. CT90 was defined as the percentage of total sleep time with oxygen saturation below 90%. Because CT90 was available only for PSG-derived reports in the current dataset, analyses involving CT90 were restricted to the corresponding PSG complete-case sample.

### 2.3 Data collection

Clinical variables were obtained from electronic medical records, including age, sex, height, weight, BMI, smoking history, and documented comorbidities. Available laboratory data included fasting glucose, triglycerides, HbA1c, total cholesterol, high-density lipoprotein cholesterol, low-density lipoprotein cholesterol, uric acid, hemoglobin, white blood cell count, and platelet count. Fasting glucose and triglyceride measurements used to calculate TyG were obtained during the same hospitalization and within 3 days before or after sleep monitoring.

Comorbidities were analyzed according to the information recorded in the medical record. Blank comorbidity fields were not assumed to indicate absence of disease and were instead treated as missing. For this reason, the denominators for hypertension and diabetes/glucose metabolism disorder were smaller than the total cohort. The diagnosis of diabetes/glucose metabolism disorder was used as a clinical comorbidity covariate rather than as a replacement for the fasting glucose component of TyG; its inclusion in the fully adjusted model was intended to explore whether the association between TyG and sleep-related outcomes persisted beyond documented metabolic disease status.

### 2.4 Calculation of the TyG index

The TyG index was calculated using the standard formula: ln[fasting triglycerides (mg/dL) × fasting glucose (mg/dL)/2]. Triglycerides measured in mmol/L were converted to mg/dL by multiplying by 88.57, and fasting glucose measured in mmol/L was converted to mg/dL by multiplying by 18.

### 2.5 Grouping strategy and outcomes

Patients were classified by nocturnal oxygenation and OSA severity. The primary oxygenation-related outcome was lowest SpO_2_ <80%, which was selected as a clinically recognizable threshold for severe nocturnal oxygen desaturation. This cutoff is supported by the 2025 Chinese guideline for adult OSA, which recommends using nocturnal lowest pulse oxygen saturation as an auxiliary measure for assessing nocturnal hypoxemia severity and classifies LSpO_2_ <80% as severe oxygen desaturation (24). This threshold was not intended to represent a formally defined sleep-apnea-specific hypoxic burden metric.

Severe AHI-defined OSA was defined as AHI ≥30 events/h. To examine graded patterns, patients with complete TyG, lowest SpO_2_, and AHI data were divided into TyG tertiles. CT90 above the median and lowest SpO_2_ <85% were analyzed as additional oxygenation-related outcomes.

### 2.6 Statistical analysis

Analyses were performed with R version 4.4.2 or equivalent validated statistical software. Continuous variables were checked for distribution and are reported as mean ± standard deviation or median (interquartile range), as appropriate. Because most core variables were skewed, medians and interquartile ranges are used for the main descriptive summaries. Categorical variables are reported as numbers and percentages. Between-group comparisons used the independent-samples t test or Mann-Whitney U test for continuous variables and the chi-square test or Fisher’s exact test for categorical variables, as appropriate.

Spearman correlation analysis was used to examine relationships between the TyG index and sleep-monitoring parameters. ROC analysis evaluated TyG for identifying lowest SpO_2_ <80%, AHI ≥30 events/h, CT90 above the median, and lowest SpO_2_ <85%. AUC 95% confidence intervals were estimated from 10, 000 bootstrap resamples, and optimal cutoffs were selected by the Youden index.

Logistic regression was used to evaluate the association of TyG with oxygenation-related outcomes or severe AHI-defined OSA. Model 1 was unadjusted; Model 2 adjusted for age, sex, and BMI; and Model 3 additionally adjusted for documented hypertension and diabetes/glucose metabolism disorder. Fasting glucose and triglycerides were not entered together with TyG in multivariable models because they are components of the index.

Additional sensitivity analyses were performed in response to the potential influence of sleep-monitoring heterogeneity and overall OSA severity. First, logistic regression models were further adjusted for monitoring modality (PSG vs. Nox T3). Second, models for lowest SpO_2_ <80% and CT90 above the median were additionally adjusted for AHI to explore whether TyG was associated with oxygenation-related outcomes independently of overall event frequency. Third, modality-stratified analyses and TyG × monitoring-modality interaction tests were performed for the two primary binary outcomes. Missing data were handled using complete-case analysis for each endpoint, and patients included in the main complete-case analysis were compared with those excluded because of missing TyG, AHI, or lowest SpO_2_ data. Two-sided P values <0.05 were considered statistically significant.

## 3 Results

### 3.1 Patient flow, monitoring modality, and missing data

Among 154 consecutive symptomatic adult inpatients screened from 2021 to 2025, 11 were excluded because of previous malignancy (n=3) or prior CPAP treatment with readmission for further care (n=8). The final retrospective dataset included 143 patients. Of these, 121 had valid in-hospital diagnostic sleep monitoring, including 89 examined by PSG and 32 examined by Nox T3 portable monitoring; 22 had no valid in-hospital diagnostic sleep-monitoring record, had only pressure-titration data, or had outside-hospital diagnostic records unavailable in the electronic medical record. These 22 patients were not included in analyses requiring AHI or oxygenation outcomes. The patient flow is shown in **Supplementary Figure 1**, and monitoring-modality distributions are summarized in **Supplementary Table 1**.

TyG could be calculated in 116 patients; 101 patients had complete TyG, lowest SpO_2_, and AHI data and constituted the main complete-case sample. Within this main sample, 77 patients underwent PSG and 24 underwent Nox T3 portable monitoring. CT90 with TyG was available in 77 patients, all of whom had PSG-derived CT90 data. Variable-level missingness is shown in **Table 1** and **Supplementary Table 2**. Compared with patients excluded from the main complete-case analysis, included patients had broadly similar age, BMI, fasting glucose, triglycerides, TyG, HbA1c, AHI, mean SpO_2_, CT90, and comorbidity distributions, although lowest SpO_2_ was lower and white blood cell count was slightly higher in the included group (**Supplementary Table 2**).

**Table 1:** Baseline characteristics and missingness of the study population.

Variable	Overall	Available n	Missing n	Missing, %
Age, years	49.000 (38.500, 58.000)	143	0	0.0
Male sex	115 (81.0%)	142	1	0.7
BMI, kg/m²	29.395 (26.498, 32.533)	139	4	2.8
Hypertension	80 (84.2%)	95	48	33.6
Diabetes/glucose metabolism disorder	29 (30.5%)	95	48	33.6
Fasting glucose, mmol/L	5.600 (5.015, 6.035)	131	12	8.4
Triglycerides, mmol/L	2.020 (1.430, 2.800)	117	26	18.2
TyG index	9.122 (8.768, 9.406)	116	27	18.9
HbA1c, %	6.100 (5.800, 6.700)	90	53	37.1
AHI, events/h	54.400 (25.600, 73.000)	121	22	15.4
Mean SpO_2_, %	93.000 (90.000, 95.000)	121	22	15.4
Lowest SpO_2_, %	69.000 (58.000, 81.000)	121	22	15.4
CT90	0.256 (0.042, 0.441)	89	54	37.8
Longest apnea duration, s	62.750 (47.700, 80.225)	120	23	16.1
White blood cells, ×10^9^/L	6.700 (5.645, 7.700)	132	11	7.7
Hemoglobin, g/L	150.000 (140.750, 157.925)	132	11	7.7
Platelets, ×10^9^/L	225.500 (185.000, 260.775)	132	11	7.7

Values are presented as median (interquartile range) or n (%). Missingness was calculated relative to the total cohort (n=143). Comorbidity variables were analyzed among patients with documented comorbidity records; blank entries were treated as missing.

### 3.2 Baseline characteristics of the study population

The final retrospective dataset included 143 patients with OSA-related evaluation. Median age was 49.0 years, and 115 of 142 patients with available sex data were male (81.0%). Median BMI was 29.395 kg/m². Baseline characteristics and variable-level missingness are shown in **Table 1**.

### 3.3 Clinical characteristics according to nocturnal oxygen desaturation severity

Using lowest SpO_2_ <80% as the threshold for severe nocturnal oxygen desaturation, patients below this threshold had a higher TyG index than those with lowest SpO_2_ ≥80% [9.252 (8.872, 9.577) vs. 8.780 (8.574, 8.963), P<0.001]. They also had higher BMI, fasting glucose, triglycerides, HbA1c, AHI, and CT90, as well as lower mean SpO_2_ and longer apnea duration (all P<0.05). **Table 2** provides the full comparison.

**Table 2:** Clinical characteristics according to lowest SpO_2_ group.

Variable	Lowest SpO_2_ ≥80% (n=32)	Lowest SpO_2_ <80% (n=89)	P value
Age, years	58.000 (43.750, 67.250)	48.000 (38.000, 56.000)	0.016
Male sex	23 (71.9%)	75 (84.3%)	0.204
BMI, kg/m²	27.639 (25.594, 29.926)	29.762 (27.042, 33.173)	0.010
Hypertension	14 (77.8%)	56 (87.5%)	0.449
Diabetes/glucose metabolism disorder	2 (11.1%)	23 (35.9%)	0.048
Fasting glucose, mmol/L	5.045 (4.540, 5.433)	5.710 (5.170, 6.265)	<0.001
Triglycerides, mmol/L	1.580 (1.370, 2.020)	2.220 (1.490, 2.933)	0.010
TyG index	8.780 (8.574, 8.963)	9.252 (8.872, 9.577)	<0.001
HbA1c, %	5.850 (5.425, 6.075)	6.200 (5.800, 6.700)	0.003
AHI, events/h	19.150 (10.800, 26.675)	62.800 (49.200, 77.000)	<0.001
Mean SpO_2_, %	95.000 (94.075, 96.075)	91.300 (89.000, 93.000)	<0.001
Lowest SpO_2_, %	85.000 (82.750, 87.000)	63.000 (54.000, 70.000)	<0.001
CT90	0.004 (0.001, 0.017)	0.315 (0.162, 0.487)	<0.001
Longest apnea duration, s	36.500 (26.225, 54.125)	71.250 (53.500, 87.875)	<0.001
White blood cells, ×10^9^/L	6.320 (5.325, 6.968)	7.075 (5.857, 8.332)	0.022
Hemoglobin, g/L	144.700 (137.500, 151.125)	152.250 (141.500, 159.750)	0.065
Platelets, ×10^9^/L	209.500 (165.750, 232.450)	227.850 (191.000, 260.925)	0.030

Values are presented as median (interquartile range) or n (%). P values were calculated using the Mann-Whitney U test, chi-square test, or Fisher’s exact test, as appropriate. Denominators differ for variables with missing data.

### 3.4 Clinical characteristics according to AHI-defined OSA severity

When patients were stratified by AHI, those with AHI ≥30 events/h had a higher TyG index than those with AHI <30 events/h [9.242 (8.869, 9.624) vs. 8.823 (8.571, 9.121), P<0.001]. The severe AHI-defined OSA group also showed higher BMI, more documented hypertension and diabetes/glucose metabolism disorder, higher fasting glucose, triglycerides, and HbA1c, lower mean and lowest SpO_2_, higher CT90, and longer apnea duration. Detailed results are listed in **Table 3**.

**Table 3:** Clinical characteristics according to AHI severity group.

Variable	AHI <30/h (n=35)	AHI ≥30/h (n=86)	P value
Age, years	56.000 (34.500, 64.500)	49.000 (39.000, 56.750)	0.194
Male sex	25 (71.4%)	73 (84.9%)	0.146
BMI, kg/m²	27.310 (25.535, 30.813)	29.410 (27.166, 33.144)	0.021
Hypertension	15 (68.2%)	55 (91.7%)	0.021
Diabetes/glucose metabolism disorder	2 (9.1%)	23 (38.3%)	0.014
Fasting glucose, mmol/L	5.030 (4.590, 5.530)	5.740 (5.220, 6.287)	<0.001
Triglycerides, mmol/L	1.610 (1.320, 2.295)	2.160 (1.490, 2.945)	0.018
TyG index	8.823 (8.571, 9.121)	9.242 (8.869, 9.624)	<0.001
HbA1c, %	5.900 (5.400, 6.100)	6.200 (5.800, 6.700)	0.006
AHI, events/h	17.600 (9.550, 24.150)	65.250 (52.200, 77.225)	<0.001
Mean SpO_2_, %	95.000 (95.000, 96.350)	91.100 (89.000, 93.000)	<0.001
Lowest SpO_2_, %	83.000 (79.000, 87.000)	63.000 (54.000, 70.750)	<0.001
CT90	0.004 (0.002, 0.016)	0.321 (0.146, 0.492)	<0.001
Longest apnea duration, s	37.600 (26.150, 57.850)	71.000 (53.500, 85.300)	<0.001
White blood cells, ×10^9^/L	6.320 (5.325, 7.078)	6.900 (5.885, 8.277)	0.045
Hemoglobin, g/L	144.200 (131.925, 155.150)	151.100 (143.000, 159.750)	0.039
Platelets, ×10^9^/L	214.000 (175.250, 236.300)	227.850 (186.000, 260.475)	0.119

Values are presented as median (interquartile range) or n (%). P values were calculated using the Mann-Whitney U test, chi-square test, or Fisher’s exact test, as appropriate. Denominators differ for variables with missing data.

### 3.5 TyG tertile analysis

**Figure 1:**
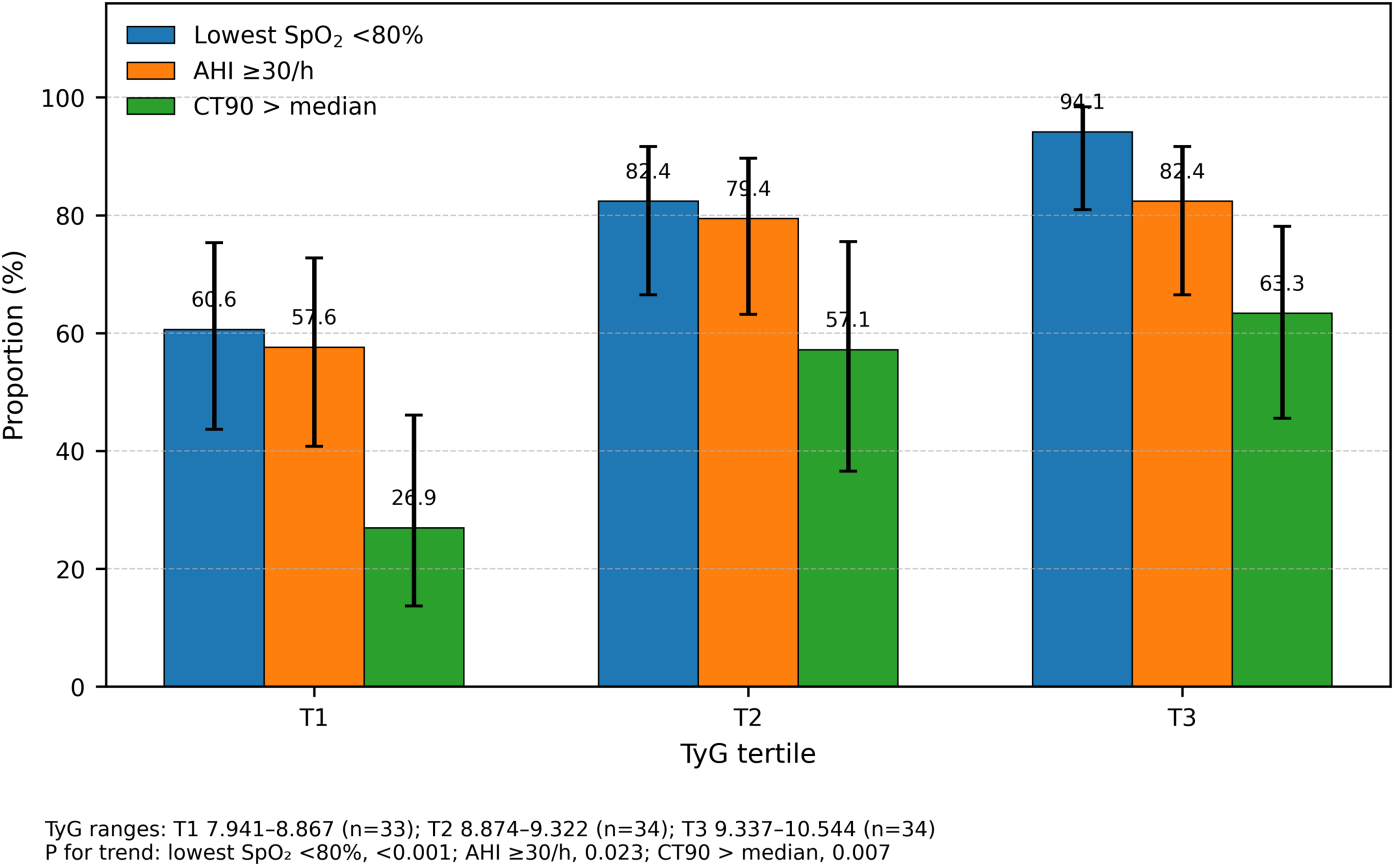
Proportions of nocturnal oxygenation and OSA severity outcomes across TyG tertiles. TyG tertiles were generated among patients with complete TyG, lowest SpO_2_, and AHI data (n=101). Error bars indicate Wilson 95% confidence intervals for proportions. P values indicate trend across increasing TyG tertiles.

Among the 101 patients with complete TyG, lowest SpO_2_, and AHI data, adverse sleep-related outcomes became more frequent across increasing TyG tertiles (**Figure 1**). The proportions of lowest SpO_2_ <80% were 60.6%, 82.4%, and 94.1% in T1, T2, and T3, respectively (P for trend <0.001). The corresponding proportions of AHI ≥30 events/h were 57.6%, 79.4%, and 82.4% (P for trend=0.023). Among patients with available CT90 data, the proportions of CT90 above the median were 26.9%, 57.1%, and 63.3% (P for trend=0.007).

### 3.6 Correlation between the TyG index and sleep-monitoring parameters

**Figure 2:**
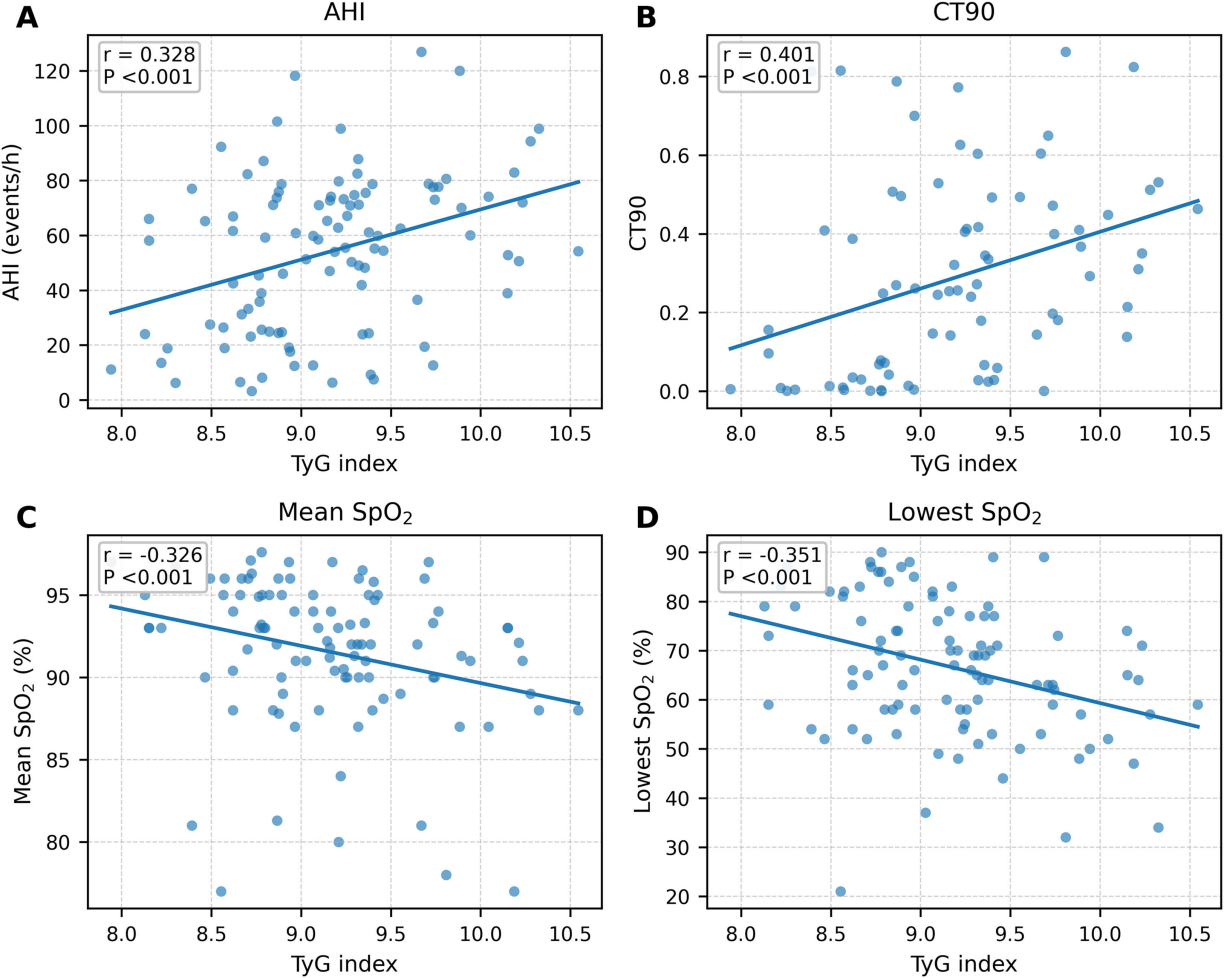
Correlations between the TyG index and sleep-monitoring parameters. **(A)** AHI; **(B)** CT90; **(C)** mean SpO_2_; **(D)** lowest SpO_2_. Spearman correlation coefficients and P values are shown in each panel.

TyG showed a positive correlation with AHI (r=0.328, P<0.001) and CT90 (r=0.401, P<0.001), and an inverse correlation with mean SpO_2_ (r=-0.326, P<0.001) and lowest SpO_2_ (r=-0.351, P<0.001). Its correlation with longest apnea duration was weak and not statistically significant (r=0.162, P = 0.107). The correlation findings are summarized in **Table 4** and illustrated in **Figure 2**.

**Table 4:** Spearman correlations between the TyG index and sleep-monitoring parameters.

Variable	n	Spearman r	P value
AHI, events/h	101	0.328	<0.001
Mean SpO_2_, %	101	-0.326	<0.001
Lowest SpO_2_, %	101	-0.351	<0.001
CT90	77	0.401	<0.001
Longest apnea duration, s	100	0.162	0.107

TyG, triglyceride-glucose index; AHI, apnea-hypopnea index; CT90, proportion of total sleep time with oxygen saturation below 90%.

### 3.7 ROC analysis

**Figure 3:**
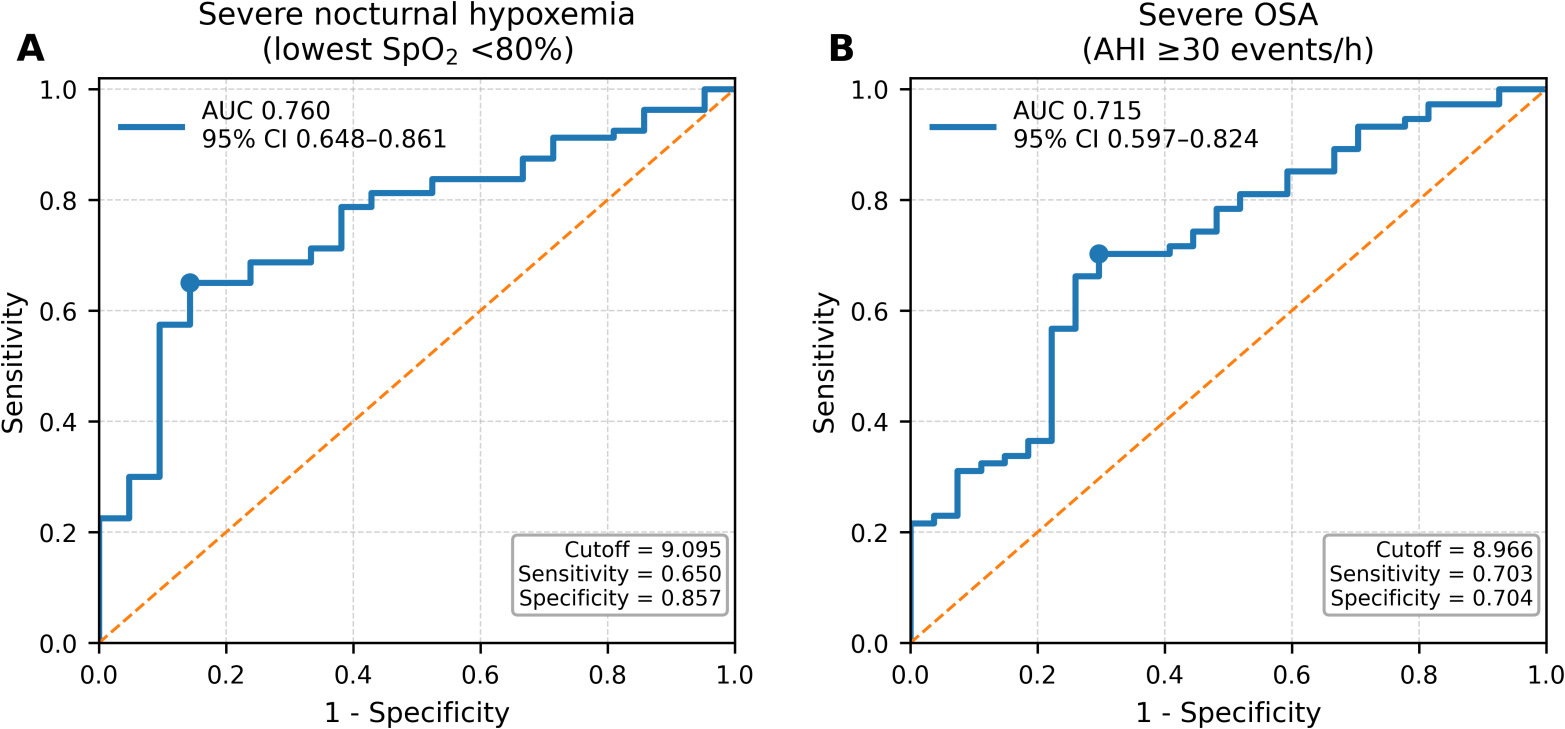
ROC curves of the TyG index for identifying severe nocturnal oxygen desaturation and severe AHI-defined OSA. **(A)** lowest SpO_2_ <80%; **(B)** AHI ≥30 events/h. AUC values are shown with bootstrap 95% confidence intervals. The marked point indicates the Youden-index optimal cutoff.

The TyG index provided moderate discrimination for both lowest SpO_2_ <80% and severe AHI-defined OSA. For lowest SpO_2_ <80%, the AUC was 0.760 (95% CI, 0.648–0.861); the Youden-index cutoff was 9.095, with sensitivity of 0.650 and specificity of 0.857. For AHI ≥30 events/h, the AUC was 0.715 (95% CI, 0.597–0.824); the cutoff was 8.966, with sensitivity of 0.703 and specificity of 0.704. The AUCs for CT90 above the median and lowest SpO_2_ <85% were 0.718 and 0.696, respectively. **Table 5** summarizes the ROC results, and **Figure 3** shows the ROC curves for the two primary outcomes. These ROC results should be interpreted as exploratory risk-stratification findings rather than diagnostic evidence.

**Table 5:** ROC analysis of the TyG index for identifying nocturnal oxygen desaturation and severe AHI-defined OSA.

Outcome	Events/n	AUC	95% CI	Optimal cutoff	Sensitivity	Specificity
Lowest SpO_2_ < 80%	80/101	0.760	0.648–0.861	9.095	0.650	0.857
AHI ≥ 30/h	74/101	0.715	0.597–0.824	8.966	0.703	0.704
CT90 > median	38/77	0.718	0.596–0.828	8.844	0.895	0.487
Lowest SpO_2_ < 85%	89/101	0.696	0.534–0.838	8.966	0.652	0.833

AUC, area under the receiver operating characteristic curve; CI, confidence interval; OSA, obstructive sleep apnea. AUC 95% CIs were estimated using 10, 000 bootstrap resamples.

### 3.8 Logistic regression and sensitivity analyses

In logistic regression, each 1-unit increase in TyG was associated with higher odds of lowest SpO_2_ <80% in the unadjusted model (OR = 7.314, 95% CI, 2.211–24.194, P = 0.001). The association persisted after adjustment for age, sex, and BMI (OR = 6.878, 95% CI, 1.960–24.130, P = 0.003). After further adjustment for documented hypertension and diabetes/glucose metabolism disorder, the estimate remained in the same direction but did not reach statistical significance (OR = 4.235, 95% CI, 0.879–20.399, P = 0.072).

TyG was also associated with AHI ≥30 events/h in the unadjusted model (OR = 5.821, 95% CI, 2.023–16.751, P = 0.001), in the age-, sex-, and BMI-adjusted model (OR = 5.787, 95% CI, 1.926–17.388, P = 0.002), and after additional adjustment for documented hypertension and diabetes/glucose metabolism disorder (OR = 7.201, 95% CI, 1.104–46.954, P = 0.039). However, the fully adjusted AHI model had a wide confidence interval and should be interpreted cautiously because of the reduced sample size and sparse events after inclusion of comorbidity variables. For CT90 above the median, the association was significant in all three models. Full regression results are provided in **Table 6**.

**Table 6:** Logistic regression analysis of the association between the TyG index and nocturnal oxygenation-related outcomes or severe AHI-defined OSA.

Outcome	Model	n	OR per 1-unit TyG	95% CI	P value
Lowest SpO_2_ < 80%	Model 1	101	7.314	2.211–24.194	0.001
Lowest SpO_2_ < 80%	Model 2	101	6.878	1.960–24.130	0.003
Lowest SpO_2_ < 80%	Model 3	70	4.235	0.879–20.399	0.072
AHI ≥ 30/h	Model 1	101	5.821	2.023–16.751	0.001
AHI ≥ 30/h	Model 2	101	5.787	1.926–17.388	0.002
AHI ≥ 30/h	Model 3	70	7.201	1.104–46.954	0.039
CT90 > median	Model 1	77	4.377	1.740–11.010	0.002
CT90 > median	Model 2	77	5.019	1.715–14.688	0.003
CT90 > median	Model 3	51	8.600	1.708–43.311	0.009

Model 1: unadjusted. Model 2: adjusted for age, sex, and BMI. Model 3: additionally adjusted for documented hypertension and diabetes/glucose metabolism disorder. TyG index was entered as a continuous variable. OR, odds ratio; CI, confidence interval. The fully adjusted AHI ≥30 events/h model should be interpreted cautiously because of sparse data and wide confidence intervals.

In sensitivity analyses adjusted for monitoring modality in addition to age, sex, and BMI, the association between TyG and lowest SpO_2_ <80% remained significant (OR = 7.166, 95% CI, 1.947–26.381, P = 0.003), as did the association between TyG and AHI ≥30 events/h (OR = 5.913, 95% CI, 1.859–18.806, P = 0.003) (**Supplementary Table 3**). Modality-stratified analyses and TyG × monitoring-modality interaction tests are presented in **Supplementary Tables 4B, C**; the interaction tests were not statistically significant for lowest SpO_2_ <80% (P for interaction=0.212) or AHI ≥30 events/h (P for interaction=0.254), although subgroup analyses were limited by the small Nox T3 sample size.

After additional adjustment for AHI, the association between TyG and lowest SpO_2_ <80% was attenuated and no longer statistically significant (OR = 3.390, 95% CI, 0.648–17.748, P = 0.148). The association between TyG and CT90 above the median also attenuated and approached but did not reach statistical significance (OR = 4.433, 95% CI, 0.977–20.111, P = 0.054) (**Supplementary Table 3**). In linear models, AHI adjustment similarly attenuated the associations of TyG with lowest SpO_2_, mean SpO_2_, and CT90 (**Supplementary Table 4A**). These findings suggest that the relationship between TyG and nocturnal oxygenation-related parameters may partly reflect overall AHI-defined OSA severity.

## 4 Discussion

In this exploratory retrospective study of hospitalized patients with OSA-related evaluation, a higher TyG index was associated with worse conventional nocturnal oxygenation-related parameters and greater AHI-defined OSA severity. TyG was higher in patients whose lowest SpO_2_ fell below 80% and in those with AHI ≥30 events/h. It also correlated positively with AHI and CT90 and inversely with mean and lowest SpO_2_, suggesting that this glucose-lipid metabolic marker is related to several sleep-monitoring indices reflecting both respiratory-event frequency and nocturnal oxygenation impairment.

The main contribution of this study is its focus on conventional oxygenation-related measures rather than AHI-defined severity alone. Prior studies have already reported associations between TyG or TyG-related indices and OSA risk or AHI-defined OSA severity (15–21). The present analysis extends this literature by examining lowest SpO_2_, CT90, and mean SpO_2_ in addition to AHI. This distinction is clinically relevant because AHI counts respiratory events but does not fully capture the depth or duration of oxygen desaturation. Contemporary OSA literature increasingly emphasizes the need to consider oxygenation-related metrics and formally defined hypoxic burden measures when evaluating physiological stress and cardiometabolic risk (7–10).

The observed associations are biologically plausible but should not be interpreted as causal. TyG combines fasting glucose and triglyceride levels and is often used as a practical marker related to insulin resistance (12–14). OSA-related intermittent hypoxemia and sleep fragmentation may promote sympathetic activation, oxidative stress, inflammation, and adipose tissue dysfunction, thereby contributing to glucose-lipid metabolic abnormalities (6, 11). Conversely, obesity, insulin resistance, dyslipidemia, and glucose metabolism disorders may also contribute to OSA pathophysiology and nocturnal oxygenation impairment. Therefore, the relationship between TyG and oxygenation-related parameters is likely bidirectional and confounded by shared cardiometabolic risk factors.

A key issue raised by the additional analyses is the role of AHI-defined OSA severity. When models were additionally adjusted for AHI, the associations of TyG with lowest SpO_2_ <80% and CT90 above the median were attenuated. This finding suggests that TyG may not be independently associated with nocturnal oxygenation impairment after fully accounting for respiratory-event frequency, or that AHI lies on the pathway linking metabolic dysfunction and oxygenation-related abnormalities. Given the cross-sectional design, these possibilities cannot be separated. Accordingly, the findings should be regarded as hypothesis-generating and supportive of further research rather than evidence that TyG independently predicts nocturnal oxygenation impairment.

The ROC analyses also require cautious interpretation. TyG showed only moderate discrimination for lowest SpO_2_ <80% and AHI ≥30 events/h. Because fasting glucose and triglycerides are routinely measured, TyG may provide a simple metabolic signal that identifies patients who merit closer evaluation of sleep-related oxygenation and cardiometabolic risk. However, the AUC values observed here are not sufficient to support TyG as a diagnostic tool or a substitute for PSG or validated portable sleep monitoring.

This study used conventional oxygenation-related parameters, not a formally calculated sleep-apnea-specific hypoxic burden metric. CT90 captures the cumulative proportion of time spent below 90% oxygen saturation, while lowest SpO_2_ reflects the nadir desaturation and is more vulnerable to artifact and night-to-night variability. By contrast, formally defined hypoxic burden quantifies the area under desaturation curves linked to respiratory events and may better reflect event-specific physiological stress (9, 10). We therefore revised the terminology throughout the manuscript to refer primarily to nocturnal oxygenation impairment or oxygenation-related parameters. Future studies should examine whether TyG is associated with formally defined hypoxic burden metrics and whether such associations predict cardiometabolic outcomes.

Several limitations should be emphasized. First, the retrospective cross-sectional design precludes causal inference and makes the results exploratory. Second, this was a single-center inpatient study. Hospitalized patients who underwent OSA-related evaluation may have more symptoms, comorbidities, or more severe disease than community-based or outpatient populations, limiting generalizability. Third, the sample size was modest, and the number of complete cases varied across endpoints because of missing TyG, CT90, and comorbidity data. The comparison between included and excluded patients suggested broadly similar characteristics, but lower lowest SpO_2_ in the included group indicates possible selection bias toward more severe oxygenation impairment. Fourth, CT90 was available only in PSG-derived reports, limiting CT90 analyses and preventing full modality-specific evaluation of this outcome. Fifth, PSG and Nox T3 portable monitoring were both used. Although monitoring-modality-adjusted and interaction analyses did not suggest a statistically significant modification of the main associations, the Nox T3 subgroup was small and estimates were imprecise. Sixth, lowest SpO_2_ and longest apnea duration are extreme-value metrics and may be influenced by signal quality, although raw sleep-monitoring and oximetry signals were manually reviewed and obvious artifacts were excluded. Seventh, fasting insulin was not available, so TyG could not be compared with HOMA-IR. Finally, medication use, diet, physical activity, and other metabolic modifiers were not fully captured. Larger prospective studies using standardized sleep monitoring, formal hypoxic burden measures, and more complete metabolic profiling are needed.

## 5 Conclusion

In this exploratory retrospective study of hospitalized patients with OSA-related evaluation, a higher TyG index was associated with worse nocturnal oxygenation-related parameters and greater AHI-defined OSA severity. However, the associations with oxygenation-related outcomes were attenuated after adjustment for AHI, suggesting that the relationship may partly reflect overall OSA severity. TyG may serve as a simple metabolic clue for identifying patients who warrant careful sleep and cardiometabolic assessment, but it should complement rather than replace formal sleep monitoring. These findings require confirmation in larger prospective studies using standardized sleep monitoring and formally defined hypoxic burden metrics.

## Data Availability

The raw data supporting the conclusions of this article will be made available by the authors, without undue reservation.
