# Supplementary material for: Triglyceride-glucose index and nocturnal oxygenation impairment in patients with obstructive sleep apnea
Source: Front Endocrinol (Lausanne). 2026 Jul 2;17:1899452. doi: 10.3389/fendo.2026.1899452 (PMC13372581; doi:10.3389/fendo.2026.1899452)
Supplement: Supplementary file 1 [file DataSheet1.docx]

**Supplementary Material**

**For**

**Triglyceride-Glucose Index and Nocturnal Oxygenation Impairment in Patients With Obstructive Sleep Apnea**

**Supplementary Figure 1. Study flow diagram of the retrospective cohort and analytic samples.**


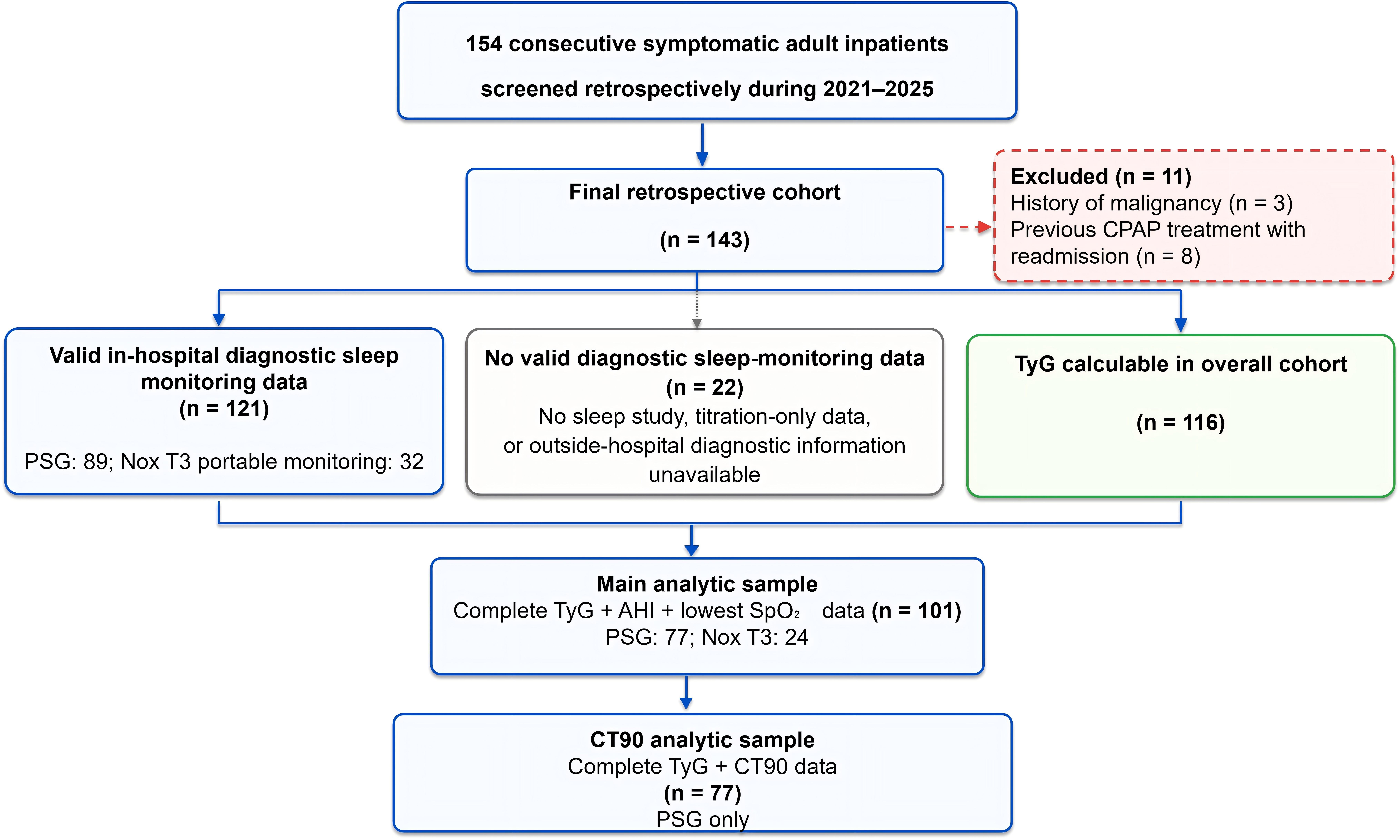


**Abbreviations:** TyG, triglyceride-glucose index; AHI, apnea-hypopnea index; PSG, polysomnography; CPAP, continuous positive airway pressure; CT90, percentage/time of oxygen saturation below 90%.

**Supplementary Table S1.** Distribution of sleep-monitoring modality.

| **Sample** | **Monitoring modality** | **n** | **%** |
| --- | --- | --- | --- |
| Overall retrospective dataset (n=143) | 1 PSG (Philips Alice 6) | 89 | 62.2% |
| Overall retrospective dataset (n=143) | 2 Portable monitoring (Nox T3) | 32 | 22.4% |
| Overall retrospective dataset (n=143) | 0 No valid in-hospital diagnostic monitoring/titration-only or outside record unavailable | 22 | 15.4% |
| Main complete-case analysis (TyG + AHI + lowest SpO₂, n=101) | 1 PSG (Philips Alice 6) | 77 | 76.2% |
| Main complete-case analysis (TyG + AHI + lowest SpO₂, n=101) | 2 Portable monitoring (Nox T3) | 24 | 23.8% |
| CT90 + TyG analysis sample (n=77) | 1 PSG (Philips Alice 6) | 77 | 100.0% |

PSG, polysomnography. Nox T3 refers to portable monitoring with Nox T3 Sleep Monitor. Patients without valid in-hospital diagnostic monitoring, pressure-titration-only data, or outside-hospital diagnostic records unavailable in the electronic record were excluded from analyses requiring AHI or oxygenation outcomes.

**Supplementary Table S2.** Comparison between patients included in and excluded from the main complete-case analysis.

| **Variable** | **Included in main analysis**  **(n=101)** | **Available n included** | **Excluded from main analysis (n=42)** | **Available n excluded** | **P value** |
| --- | --- | --- | --- | --- | --- |
| Age, years | 49.000  (37.000, 57.000) | 101 | 50.000 (40.250, 62.750) | 42 | 0.301 |
| BMI, kg/m² | 29.395  (26.028, 32.050) | 101 | 29.903 (27.266, 32.555) | 38 | 0.574 |
| Fasting glucose, mmol/L | 5.570 (5.030, 5.970) | 101 | 5.635 (4.982, 6.130) | 30 | 0.932 |
| Triglycerides, mmol/L | 2.050 (1.440, 2.880) | 101 | 1.880 (1.225, 2.320) | 16 | 0.396 |
| TyG index | 9.161 (8.780, 9.405) | 101 | 8.877 (8.638, 9.317) | 15 | 0.325 |
| HbA1c, % | 6.100 (5.800, 6.600) | 71 | 6.200 (5.850, 7.000) | 19 | 0.517 |
| AHI, events/h | 58.500 (26.400, 74.100) | 101 | 49.600 (19.175, 56.850) | 20 | 0.063 |
| Mean SpO₂, % | 92.100 (90.000, 95.000) | 101 | 93.650 (91.300, 95.000) | 20 | 0.224 |
| Lowest SpO₂, % | 67.000 (58.000, 78.000) | 101 | 80.500 (62.750, 85.250) | 20 | 0.047 |
| CT90 | 0.256 (0.059, 0.463) | 77 | 0.189 (0.016, 0.296) | 12 | 0.285 |
| Longest apnea duration, s | 64.000 (49.375, 80.225) | 100 | 48.900 (38.675, 81.975) | 20 | 0.326 |
| White blood cells, ×10⁹/L | 6.850 (5.780, 7.860) | 97 | 6.130 (5.345, 7.160) | 35 | 0.041 |
| Hemoglobin, g/L | 151.000 (140.000, 159.000) | 97 | 149.000 (141.000, 155.000) | 35 | 0.374 |
| Platelets, ×10⁹/L | 226.000 (186.000, 257.100) | 97 | 214.000 (172.500, 267.500) | 35 | 0.42 |
| Male sex | 84 (83.2%) | 101 | 31 (73.8%) | 42 | 0.292 |
| Hypertension | 60 (85.7%) | 70 | 20 (80.0%) | 25 | 0.724 |
| Diabetes/glucose metabolism disorder | 22 (31.4%) | 70 | 7 (28.0%) | 25 | 0.947 |

Main complete-case analysis was defined as availability of TyG index, AHI, and lowest SpO₂. Values are median (interquartile range) or n (%). P values were calculated using Mann-Whitney U, chi-square, or Fisher's exact tests, as appropriate.

**Supplementary Table S3.** Sensitivity logistic regression analyses adjusted for monitoring modality or AHI.

| **Outcome** | **Model** | **n** | **Events** | **OR (95% CI)** | **P value** |
| --- | --- | --- | --- | --- | --- |
| Lowest SpO₂ <80% | Age sex BMI + monitoring modality | 101 | 80 | 7.166 (1.947–26.381) | 0.003 |
| AHI ≥30 events/h | Age sex BMI + monitoring modality | 101 | 74 | 5.913 (1.859–18.806) | 0.003 |
| Lowest SpO₂ <80% | Age sex BMI + AHI | 101 | 80 | 3.390 (0.648–17.748) | 0.148 |
| CT90 > median | Age sex BMI + AHI | 77 | 38 | 4.433 (0.977–20.111) | 0.054 |

TyG index was entered as a continuous variable. Monitoring-modality-adjusted models adjusted for age, sex, BMI, and monitoring modality. AHI-adjusted models adjusted for age, sex, BMI, and AHI.

**Supplementary Table S4.** Additional sensitivity analyses including AHI-adjusted linear models, modality-stratified analyses, and interaction tests.

**A. AHI-adjusted linear models**

| **Outcome** | **Model** | **n** | **β (95% CI)** | **P value** | **R²** |
| --- | --- | --- | --- | --- | --- |
| Lowest SpO₂, % | Age, sex, BMI | 101 | -7.964 (-12.563–-3.364) | <0.001 | 0.249 |
| Lowest SpO₂, % | Age, sex, BMI, AHI | 101 | -2.992 (-6.960–0.976) | 0.138 | 0.513 |
| Mean SpO₂, % | Age, sex, BMI | 101 | -2.013 (-3.405–-0.621) | 0.005 | 0.32 |
| Mean SpO₂, % | Age, sex, BMI, AHI | 101 | -0.528 (-1.737–0.681) | 0.388 | 0.553 |
| CT90 | Age, sex, BMI | 77 | 0.130 (0.054–0.207) | 0.001 | 0.435 |
| CT90 | Age, sex, BMI, AHI | 77 | 0.044 (-0.020–0.108) | 0.174 | 0.666 |

**B. Modality-stratified logistic models**

| **Outcome** | **Modality** | **Model** | **n** | **Events** | **OR (95% CI)** | **P value** |
| --- | --- | --- | --- | --- | --- | --- |
| Lowest SpO₂ <80% | PSG (Philips Alice 6) | Unadjusted | 77 | 64.0 | 11.478 (2.510–52.484) | 0.002 |
| Lowest SpO₂ <80% | PSG (Philips Alice 6) | Age sex BMI | 77 | 64.0 | 12.828 (2.454–67.045) | 0.002 |
| AHI ≥30 events/h | PSG (Philips Alice 6) | Unadjusted | 77 | 61.0 | 7.882 (2.147–28.929) | 0.002 |
| AHI ≥30 events/h | PSG (Philips Alice 6) | Age sex BMI | 77 | 61.0 | 8.631 (2.139–34.826) | 0.002 |
| Lowest SpO₂ <80% | Portable monitoring (Nox T3) | Unadjusted | 24 | 16.0 | 1.758 (0.117–26.506) | 0.684 |
| Lowest SpO₂ <80% | Portable monitoring (Nox T3) | Age sex BMI | 24 | 16.0 | 1.350 (0.070–26.002) | 0.842 |
| AHI ≥30 events/h | Portable monitoring (Nox T3) | Unadjusted | 24 | 13.0 | 1.671 (0.124–22.465) | 0.699 |
| AHI ≥30 events/h | Portable monitoring (Nox T3) | Age sex BMI | 24 | 13.0 | 1.383 (0.088–21.736) | 0.818 |

**C. Interaction tests**

| **Outcome** | **n** | **Events** | **Interaction term** | **P for interaction** |
| --- | --- | --- | --- | --- |
| Lowest SpO₂ <80% | 101 | 80.0 | TyG × Nox T3 modality | 0.212 |
| AHI ≥30 events/h | 101 | 74.0 | TyG × Nox T3 modality | 0.254 |

Linear models report beta coefficients for TyG. Stratified logistic models report ORs per 1-unit TyG. Interaction tests evaluated TyG × Nox T3 monitoring modality.
